# Simulated patient encounters to improve adolescent retention in HIV care in Kenya: study protocol of a stepped-wedge randomized controlled trial

**DOI:** 10.1186/s13063-017-2266-z

**Published:** 2017-12-28

**Authors:** Kate S. Wilson, Cyrus Mugo, David Bukusi, Irene Inwani, Anjuli D. Wagner, Helen Moraa, Tamara Owens, Joseph B. Babigumira, Barbra A. Richardson, Grace C. John-Stewart, Jennifer A. Slyker, Dalton C. Wamalwa, Pamela K. Kohler

**Affiliations:** 1 Department of Global Health, University of Washington, 325 9th Avenue, Box 359932, Seattle, WA 98104 USA; 2 Department of Paediatrics and Child Health/Kenyatta National Hospital, University of Nairobi, Nairobi, Kenya; 3 VCT and HIV Prevention Unit/Youth Centre, Kenyatta National Hospital, Nairobi, Kenya; 4 Clinical Skills and Simulation Center, Howard University Health Sciences, Washington DC, USA; 5 Department of Biostatistics, University of Washington, Seattle, WA USA; 6 Department of Pediatrics, University of Washington, Seattle, WA USA; 7 Department of Epidemiology, University of Washington, Seattle, WA USA; 8 Department of Psychosocial and Community Health, University of Washington, Seattle, WA USA; 9 Department of Medicine, University of Washington, Seattle, WA USA

**Keywords:** Adolescents and young adults, Retention in HIV care, Clinical training intervention, Standardized patients, Stepped-wedge trial

## Abstract

**Background:**

Adolescent-friendly policies aim to tailor HIV services for adolescents and young adults aged 10–24 years (AYA) to promote health outcomes and improve retention in HIV care and treatment. However, few interventions focus on improving healthcare worker (HCW) competencies and skills for provision of high-quality adolescent care. Standardized patients (SPs) are trained actors who work with HCWs in mock clinical encounters to improve clinical assessment, communication, and empathy skills. This stepped-wedge randomized controlled trial will evaluate a clinical training intervention utilizing SPs to improve HCW skills in caring for HIV-positive AYA, resulting in increased retention in care.

**Methods/design:**

The trial will utilize a stepped-wedge design to evaluate a training intervention using SPs to train HCWs in assessment, communication, and empathy skills for AYA HIV care. We will recruit 24 clinics in Kenya with an active electronic medical record (EMR) system and at least 40 adolescents enrolled in HIV care per site. Stratified randomization by county will be used to assign clinics to one of four waves – time periods when they receive the intervention – with each wave including six clinics. From each clinic, up to 10 HCWs will participate in the training intervention. SP training includes didactic sessions in adolescent health, current guidelines, communication skills, and motivational interviewing techniques. HCW participants will rotate through seven standardized SP scenarios, followed by SP feedback, group debriefing, and remote expert evaluation. AYA outcomes will be assessed using routine clinic data. The primary outcome is AYA retention in HIV care, defined as returning for first follow-up visit within 6 months of presenting to care, or returning for a first follow-up visit after re-engagement in care in AYA with a previous history of being lost to follow-up. Secondary outcomes include HCW competency scores, AYA satisfaction with care, and AYA clinical outcomes including CD4 and viral load. Additional analyses will determine cost-effectiveness of the intervention.

**Discussion:**

This trial will contribute valuable information to HIV programs in Kenya and other low-resource settings, providing a potentially scalable strategy to improve quality of care and retention in critical HIV services in this population.

**Trial registration:**

ClinicalTrials.gov, ID: NCT02928900. Registered 26 August 2016.

**Electronic supplementary material:**

The online version of this article (doi:10.1186/s13063-017-2266-z) contains supplementary material, which is available to authorized users.

## Background

Inadequate provision of accessible and acceptable HIV testing, counseling, and treatment services is a barrier to uptake of, and retention in, HIV care among adolescents and young adults aged 10–24 years (AYA) [1–3]. Studies from Africa have reported loss to follow-up from HIV care, with varying definitions, among AYA ranging from 15 to 57% [4–6]. Poor engagement in care may increase risk of HIV transmission and early death [7, 8]. Reducing HIV-related mortality and onward transmission requires the identification of evidence-based interventions that promote adolescent uptake of, and retention in, HIV care [1].

Quality of care and patient satisfaction with HIV services have been shown to affect retention in care [9–12]. In particular, AYA have expressed reluctance to seek care for fear of judgment by HCW, perceived HIV-stigma and discrimination by HCWs, and concerns over lack of confidentiality in healthcare settings [13–15]. AYA experience unique physiological, developmental, and psychosocial changes that require services appropriate to their developmental stage [16, 17]. Initiatives are underway to implement the World Health Organization guidelines for “adolescent-friendly” health services [18], with the premise that offering care that is accessible, acceptable, appropriate, equitable, and effective will improve AYA engagement in health services and clinical outcomes. Healthcare workers (HCWs) are encouraged to be “*non-judgmental* and *considerate*” and have the “*competencies to deliver the right health services in the right way*” [19]. The Kenyan government has developed ambitious guidelines for adolescent-friendly HIV services in Kenya. However, there is a gap between advertised “adolescent-friendly services” and HCWs’ reported skills and training to deliver such care [14, 20].

Standardized patients (SPs) are trained actors who work with HCWs in simulated clinical encounters for training and evaluation [21]. Clinical training using SPs is an acceptable and effective approach to improve HCW skills in patient-centered care, including empathy, communication skills, and adherence to clinical guidelines [22, 23]. Studies in low-resource settings have used SPs to evaluate quality of health service delivery [20, 24–26], but there are few examples of SP training interventions. Emerging evidence suggests that this methodology not only improves service delivery, but can also improve patient outcomes [27, 28]. Given the success of SP programs in other settings, it is plausible that high-quality, patient-centered approaches could fill this “know-do gap” in quality, and increase retention in care among HIV-positive AYA.

There is a lack of scalable, evidence-based interventions that build adolescent-friendly capacities in HCWs and that have been linked to improved AYA care outcomes [29, 30]. To address this need, the University of Nairobi and the University of Washington have partnered with the Kenya Ministry of Health’s National AIDS and STI Control Program (NASCOP) to evaluate the effectiveness of an innovative clinical training intervention targeting adherence to clinical guidelines, as well as communication and interpersonal skills among HCW.

The intervention will be evaluated using a stepped-wedge randomized controlled trial (RCT) design. We hypothesize that SP training will improve HCW competency and AYA satisfaction, increase AYA retention in HIV care, and will be cost-effective. Results from this study could demonstrate a scalable intervention to improve AYA services, retention, and programmatic impact in HIV care clinics.

## Methods/design

### Study design

**Fig. 1:**
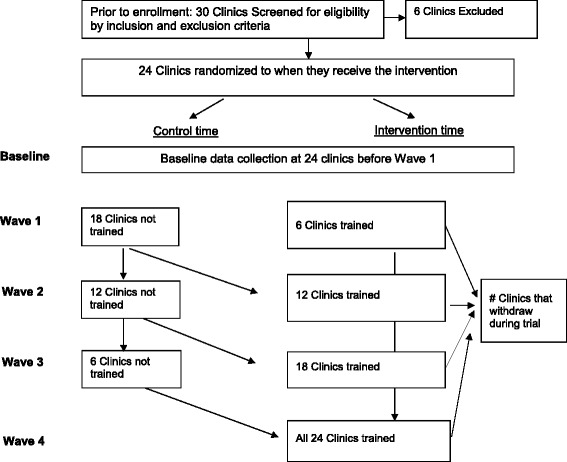
Adapted CONSORT Diagram for trial

The Simulated Patient Encounters to promote Early Detection and engagement in care for adolescents (SPEED) trial aims to evaluate the effectiveness of the SP training intervention using a stepped-wedge RCT design in 24 HIV care clinics in Kenya. This pragmatic study design was chosen because this training intervention occurs at the clinic, rather than at the individual level, uses routine clinic data for primary outcome measurement, and would not be feasible to implement simultaneously at all facilities [31]. In this modified, one-way, cross-over design, all facilities eventually receive the intervention, which was important for government partners. Facilities are randomized to *when* the intervention is introduced, which allows for benefits of randomization [32]. All facilities will contribute intervention and control periods. The RCT consists of four intervention waves approximately 9 months apart. Each wave includes HCW participants from six facilities (Fig. 1). Once facilities receive the training intervention, they are considered “exposed” until the end of the trial.

### Study sites and population

We engaged NASCOP and county leadership during the planning phase of this study to identify eligible health facilities. We also established a study Community Advisory Board (CAB), including AYA leaders from networks of people living with HIV, to provide guidance on recruitment and retention of facilities and participants. The sampling frame included secondary and tertiary HIV care facilities from four counties in Kenya that were purposefully selected to represent different HIV epidemic patterns and services to enhance generalizability by geographic region and HIV prevalence. Eligible clinics had at least 40 AYA enrolled in care, a current electronic medical record (EMR) system, and no other special adolescent interventions. Facilities were excluded if anything would prevent the complete conduct of the training intervention at that site and/or the collection of outcome measures (e.g., discontinuation of the EMR system). Among 30 eligible facilities, we selected 24 facilities ensuring a balanced distribution of counties and facility sizes in each wave.

Electronic medical records from all AYA patients ages 10–24 years enrolled in HIV care at participating sites are eligible for data abstraction. A sample of AYA presenting for HIV care at enrolled sites are further eligible to complete patient satisfaction surveys. Eligible HCW participants are 18 years of age or older, employed at trial sites, and provide clinical and/or counseling services to adolescents. Individuals are excluded if they experience any conditions that would place them at increased risk of harm or preclude the individual’s completion of the study.

### Randomization

Facilities were randomized to one of four intervention waves (Table 1), stratified by county: Nairobi, Kiambu, Kisumu, Homa Bay and by facility size. County was used for stratification because it is a reasonable marker of differences in AYA HIV prevalence and HIV care services across regions [33]. All possible combinations that yielded an allocation of two Nairobi, two Kiambu, one Homa Bay, and one Kisumu site, half high (more than 73 AYA) and half medium volume (less than 73 AYA, based on the median number of AYA enrolled in all sites) in each wave were generated in Excel. One of the possible combinations was randomly chosen using a random-number generator for the randomization allocation.

**Table 1:**
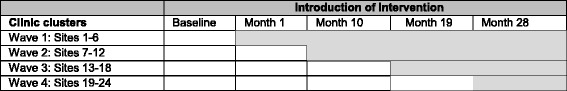
Overview of the SPEED Intervention Timing, by Wave

Key: white boxes = unexposed periods; grey boxes= exposed periods

### Blinding

Because this is a clinical training intervention using a pragmatic trial design, it is impossible to blind all members of the study team or trial participants to the randomization assignments. However, we have implemented procedures to minimize the number of individuals who are unblinded. Only the biostatistician has access to the randomization assignments of the 24 facilities. One month before each wave, the biostatistician provides a list of the six facilities in that wave to the study lead and the coordinator. All other facilities are informed that training will occur at a later wave. An independent External Advisory Panel reviews recruitment, enrollment, and potential for social harms annually during the trial. Because of the stepped-wedge design, there are no interim analyses or stopping rules.

### Training of standardized patients

We contracted with a casting agency in Kenya to recruit professional actors to be trained as SPs, hiring 14 Kenyan actors over age 18 years who could believably role-play AYA patients between 14 and 21 years. Before piloting the intervention, actors received a 5-day training facilitated by a consultant expert in the SP methodology. The training included review of seven case scripts, individualized coaching on role playing cases believably without over dramatizing, video-taped practice of the simulated patient encounters with actual HCWs followed by group discussion to refine the SPs’ portrayal of the cases, and training on providing verbal feedback to HCWs and completing the SP actor feedback forms. At the end of the training, the consultant and study team determined the final case assignments (Additional file 1).

### Recruitment and enrollment: HCWs and AYA clients

Clinic leadership personnel are consulted to provide a list of up to 10 HCWs who provide adolescent services and to negotiate release from work to participate in the training and survey data collection. A study interviewer then approaches those HCWs to invite them to learn more about the study. HCWs provide written informed consent. HCWs are tracked over time to assess provider retention at clinical intervention sites, using a facility survey to monitor staff turnover. All AYA seeking HIV care on a given day are offered referral by a clinical staff member to our study team for survey participation. We schedule facility visits to “adolescent clinic” days or during school breaks to ensure adequate volumes of eligible patients. Oral consent or assent is obtained from AYA based on their status as emancipated minors. AYA aged 18 years and older, and those aged 14–17 years who are emancipated minors, provide oral consent to participate in surveys. Otherwise, parents/caregivers provide oral informed consent for AYA ages 10–17 years, and those adolescents provide oral assent.

### SP training intervention

The SP intervention is based on an adaptation of Kolb’s model of Experiential Learning [34], Bandura’s model of Social Learning Theory [35], and an adaptation of Andersen’s Behavioral Model of Health Services Use [36]. The 2-day training intervention focuses on skill-building through practice, with a series of SP encounters, individual feedback by SPs, and group debriefings. HCW participants receive orientation to the SP methodology, instructions for taking part in the encounters, and short didactic sessions on adolescent-friendly services in Kenya and motivational interviewing techniques [37]. After the didactic session, each participant rotates through seven SP encounters. Encounters are video-recorded and occur at a training venue with stations set up to resemble counseling rooms, separated by a wall or curtain for privacy. At the end of each encounter, the SP provides non-technical, emotional feedback. Facilitated group debriefings consist of review of videos and discussion about strengths and areas for improvement in each encounter.

### Standardized case scripts

Each case script follows a standard format, with a chief complaint, relevant medical and social history, and actor prompts (Additional file 2). Qualitative analysis of in-depth interviews with HIV-positive AYA enrolled in a study on HIV-testing services in Nairobi, Kenya informed the range of cases and chief complaints [38]. We also consulted World Health Organization (WHO) and Kenyan guidelines on “adolescent-friendly” services to ensure that the cases covered major AYA health concerns, including contraception and fertility desire (whether the client wants children in the future) [5, 13], sexual identity [39], mental health [40], HIV-stigma and disclosure [14], and gender-based violence [41]. These issues also are associated with poor retention in health services [5, 13, 42, 43]. Case scripts were reviewed by Kenyan general practitioners, pediatricians, and a psychiatrist with experience in AYA care; the training consultant; and SP actors for accuracy and relevance to local context. There are seven cases in total, including both male and female roles, and one case with an older sibling present as the caregiver (Table 2).

**Table 2:** SPEED Training Intervention Case Scripts

Case Name	Key features and health concerns
Alex, Lisa (Case “0”)	17 year old male (Alex) or female (Lisa) with no complaints, good adherence, disclosed to family
Teddy	15 year old male, cognitive delay that can be mistaken for depression
Rehema	17 year old female, needs family planning, had bad experiences with health care providers in the past. She has a history of tuberculosis.
Nick, accompanied by older sister Emily	13 year old male whose is unaware of his HIV status and thinks he is taking medicine for a lung infection. He is in need of disclosure counseling. His older sister, who is also HIV-positive, is his primary caregiver. She has been hiding his HIV status from him all these years. She distrusts medical providers and HIV treatments, and sometimes gives her brother herbal treatments.
Lucy	18 year old female with STI symptoms, an older “sponsor” who is abusive, and an alcohol problem related to being in an abusive relationship.
Ian	16 year old gay-identified who is afraid of coming out to his friends and family. His HIV status is not his primary concern. He experiences suicidal ideation because he cannot be himself around anyone. For this reason, he has poor adherence and alcohol problems.
Ashley	19 year old female in need of counseling about fertility desire. She has mixed feelings about having a child. Her boyfriend knows her HIV-status and is pressuring her to get pregnant.

### Intervention waves

**Fig. 2:**
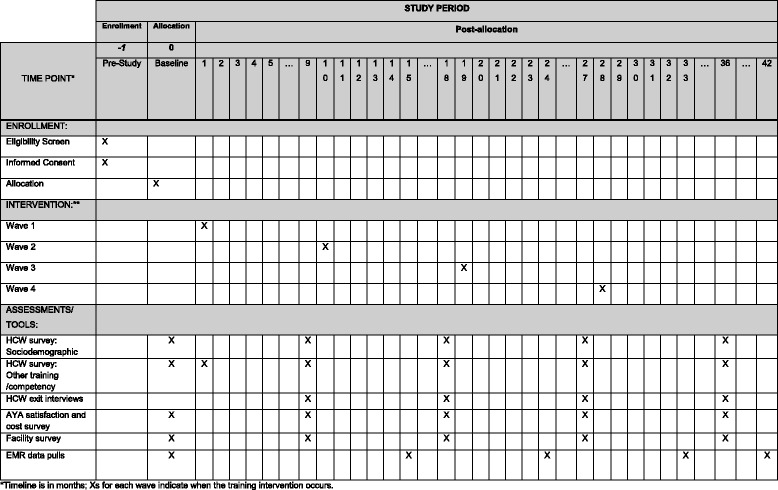
Adapted Standard Protocol Items: Recommendations for Interventional Trials (SPIRIT) Diagram for trial

All training materials and data collection tools were pilot tested. The SP training intervention is implemented at the 24 facilities over four waves (Fig. 2, Additional file 3: Standard Protocol Items: Recommendations for Interventional Trials (SPIRIT) Checklist). Each intervention wave trains HCWs from six clinics, approximately every 9 months. We anticipate that training all HCW participants in each wave will take up to 4 weeks. Each wave includes HCW participants from all four counties. We repeat the training with groups of participants from each facility or county until all participants are trained; timeframes vary based on HCW availability and travel. The intervention training schedule is planned to minimize disruption to clinic operations and reflect a “real-world” in-service training.

### Monitoring intervention fidelity

Intervention fidelity is monitored throughout the trial. The SP training included practice-simulated encounters with actual HCWs, which helped to increase the realism and relevance of the case and identify areas where the actor needed additional training. We review a random sample of video-taped encounters in each intervention wave and rate actor fidelity according to a standardized checklist that has been adapted for this study [44]. We conduct refresher trainings before each intervention wave, and follow-up refresher trainings for actors who need additional support. We use standard operating procedures and standard training materials for the didactic sessions, track HCW retention in the training intervention, and track retention of SPEED-trained HCWs at study facilities over time.

### Outcome measures using EMR data

The primary outcome for this study is AYA retention in HIV care at the individual level, defined as the probability of returning for first follow-up visit within 3 months of enrollment *or* returning for the first follow-up visit within 3 months after a “re-engagement visit,” among AYA who had been previously lost to follow-up (LTFU) (Table 3). A “re-engagement in care” visit is defined as a clinic visit after at least a 3-month absence from care. Because AYA who come for a re-engagement visit are effectively treated as new clients, this group is likely similar to newly enrolled AYA clients, and their inclusion will increase study power. Because there is no universal definition of LTFU [45] and the patterns of return visits are not well documented in AYA [4, 46], we will test an alternative definition of our primary outcome in a sensitivity analysis as return for *any* follow-up visit within 3 months among AYA currently enrolled in HIV care (i.e., patients “actively enrolled”).

**Table 3:** Summary of Primary, Process, and Exploratory Outcomes, Indicators, and Sources

	Indicator	Source
Primary Outcomes		
AYA retention in care	Follow-up visit within 3 months for newly enrolled AYA or those Lost to Follow Up (LTFU) (i.e. out of care for more than 3 months)	Clinic records
Process Outcomes		
HCW competency	Mean HCW scores per clinic	Faculty graded scores Exit interviews with trained HCWs
HCW satisfaction	Mean HCW score per clinic	HCW survey
AYA patient satisfaction	Mean AYA score per clinic	Tablet-based questionnaire
Exploratory Outcomes		
Adherence	Refills within 1 week of scheduled return visit; 95% or one or fewer missed doses in past month/dosing period	Clinic/pharmacy records
Viral suppression	Viral load (VL) <1000 copies per ml and a secondary analysis using VL <400 copies/ml	Clinic records
Linkage to APS services	STI/TB screening, Contraception uptake, mental health referral	Clinic records
Opportunistic Infections (OI)	Indication of at least one OI	Clinic records
Hospitalizations	Number of hospitalizations	Clinic records
AIDS defining illness	Diagnosis of at least one AIDS defining illness per national guidelines	Clinic record
Mortality	Death during follow up	Clinic records
Cost effectiveness and Utility Outcomes		
Cost effectiveness	Cost per additional HIV-positive AYA retained in care	Tablet-based questionnaire, published literature
Cost utility	Cost per life year saved and disability-adjusted life (DALY) averted	Tablet-based questionnaire, published literature

*APS* Adolescent Package of Services, *HCW* Healthcare Worker

### Secondary outcomes

Process and exploratory outcomes for the trial are described in Table 3. Process measures are collected using surveys to understand mechanisms of intervention effectiveness: HCW satisfaction, HCW competency, and AYA patient satisfaction with care. Exploratory outcomes to evaluate additional intervention effects on AYA outcomes are abstracted from EMR data. They include: antiretroviral therapy (ART) adherence (≥95% based on number of missed doses in past month by self-report. Refills within 1 week of scheduled return visit.); virologic suppression (<1000 copies/ml); linkage to Adolescent Package of Care services (e.g., family planning); number of hospitalizations; number of opportunistic infections; AIDS-defining illness; and mortality.

### Costing, cost-effectiveness, and cost-utility outcomes

Outcomes for the health economic analysis are: cost-effectiveness, defined as the cost per additional HIV-positive AYA retained in care and, cost-utility of the intervention, defined as cost per disability-adjusted life-year (DALY) averted and cost per life-year saved. Additional exploratory outcomes for the cost-effectiveness analysis will include: cost per additional HIV-positive AYA with good adherence as defined in the EMR (≥95% or one or fewer missed dose in past month/dosing period. Refills within 1 week of scheduled return visit); cost per additional HIV-positive AYA virally suppressed; and cost per additional HIV-positive AYA linked to adolescent package of services defined by the Kenyan Ministry of Health (e.g., referrals for mental health counseling, family planning, gender-based violence care). Cost data will be estimated from AYA surveys, program records, and published literature, and include direct medical costs, direct non-medical costs, and indirect costs.

### Data collection

Eligible de-identified AYA clinic records from the EMR system are abstracted from each facility at baseline, and at the end of each wave to assess primary and exploratory outcomes (Table 3). Data for the primary outcome will be limited to records from newly enrolled and recently re-engaged AYA clients. The time period for data abstraction is 15 months before baseline up until 15 months after the wave 1-4 training intervention. Each data abstraction period covers 15 months to allow sufficient time to observe AYA retention outcomes or approximately two school breaks (about 3–6 months apart) when AYA typically come for HIV care. Data are abstracted and transferred to an encrypted external hard drive. Survey data are collected on electronic tablets using the Open Data Kit (ODK) software (https://opendatakit.org/) [47]. The software provides an intuitive interface for validated data entry, automated export procedures for data downloads, and procedures for importing external data. Survey data are automatically uploaded to the secure study server when the device is connected to the Internet. Video-recordings are made of each HCW-SP interaction for debriefing purposes. Videos are reviewed and scored by a team of Kenyan and US-based experts to provide technical feedback on clinical competencies. Semi-structured exit interviews are conducted with a purposeful sample of up to 30 HCWs approximately 9–24 months after the SPEED intervention training to assess durability of new skills over time.

### Sample size calculation

Sample size and power calculations used methods described by Hussey and Hughes [32]. Assuming 75% of AYA in the control period will return to clinic after first visit [6], a 15% dropout without replacement, a coefficient of variation (CV) of 0.25, and a two-tailed test with *α* = 0.05, 24 clusters and five time points, we would have 80% power to detect a 15% difference, or achievement of 90% retained in care, between control and intervention periods. The lower bound of the sample size required is estimated to be 720 clinic records (6 records/site × 24 sites × 5 time points). In the optimal scenario, with an average of 40 newly enrolled AYA clients at each facility per wave, and five data collection points, our estimated sample size would be 4800 clinic records. Under the same assumptions as above, we would have > 99% power to detect a 15% difference in retention between the intervention and control periods.

### Statistical methods and analysis

The primary analysis is intent-to-treat (ITT), assuming that AYA who receive care at clinics after the SPEED training will be “exposed” to HCWs trained in the SPEED intervention until the end of the study. No replacement of facilities will be used once the trial has begun. Baseline values of primary and process outcomes, facility characteristics, and socio-demographic characteristics of AYA and HCWs will be presented in descriptive tables.

We will use generalized linear mixed models (GLMM) to compare the probability of AYA retention in HIV care between the exposed (i.e., intervention) and unexposed (i.e., control) periods [48]. To estimate the effect of the intervention (*Xij*) on the individual level, we will use a GLMM model with a binomial distribution and log link, allowing for random effects for clusters (*u*_*i*_) and fixed effects for time (*j*). This approach models individual-level outcomes, adjusts for temporal trends, and accounts for correlation of outcomes within multiple levels of clustering (clinic, individual). We will estimate adjusted risk ratios (ARR) and 95% confidence intervals at an *α* = 0.05 level (two-sided). We will estimate the effect of the intervention on each secondary outcome using GLMM to generate ARRs. All models will be adjusted for clustering and time (*c*). *The equation for a generic model is:*

$$ {Y}_{ij k}={\beta}_0+\beta {j}_{time}+{\beta}_{effect}{X}_{ij},+\beta c{X}_{ij},{u}_i+{\varepsilon}_{ij} $$

where *i* indexes the cluster (facility), *j* indexes time, and *k* indexes the individual.

We will conduct exploratory analyses to evaluate intervention mechanisms, non-constant temporal trends, and lag effects. We will examine whether an observed intervention effect may be explained through HCW post-training competency and AYA patient satisfaction scores. In that analysis, mean AYA satisfaction scores will be evaluated as predictors of AYA retention in care from EMR data. Temporal trends that change over time can confound the effect estimate in a stepped-wedge trial design [32, 48]. For example, new AYA HIV treatment guidelines may be introduced nationwide during the intervention that affect AYA retention. Time-varying temporal trends will be evaluated using methods recommended for stepped-wedge trials [48, 49]. The effect of the intervention may not be immediate or it may diminish. Intervention lag will be evaluated by including an interaction term between the intervention status (fixed effect) and time [48], and, separately, by using a fractional term for the coefficient of primary-effect estimate, which will reduce in magnitude at each time step [49].

Interview data will be analyzed using a constant comparative approach by two researchers [50]. They will code transcripts separately and compare results, resolving differences in application of codes through discussion. A codebook will be developed based on preliminary reading of the transcripts, and revised based on this iterative process. Patterns identified in the coding within and across interviews will be used to identify major themes around HCW uptake and application of skills learned in the training intervention. Results will be triangulated with the quantitative results in the study.

We will estimate the cost-effectiveness of the SP intervention in terms of cost per additional HIV-positive AYA identified and retained in care. The primary effectiveness estimate from the trial will be used for the number of additional AYA retained in care as a result of the intervention. Direct medical costs include laboratory tests, medication costs, other medical procedure costs, and personnel salaries. Direct non-medical costs include AYA transportation, food, and childcare costs. Indirect costs include opportunity costs from lost wages in AYA paid and unpaid work. We will also model the cost-utility of the intervention in terms of cost per life-year saved and DALY averted by maintaining HIV-positive AYA in care, including subsequent decreases in HIV transmission/acquisition. This will require linking effectiveness data to a mathematical model that estimates DALYs in AYA HIV progression, using a model adapted for AYA populations.

We will examine how these estimates vary through one-way and probabilistic sensitivity analyses in settings with varying AYA HIV prevalence, HCW wages, patient volume, and linkage to care rates. This will assist in identifying scenarios where the intervention is most cost-effective, allowing policy-makers to determine incremental costs and net benefits of the intervention. We will perform this analysis both from a limited societal perspective and from a Ministry of Health (healthcare sector) perspective. All benefits and costs will be discounted at 3% per year [51].

## Discussion

With global commitment to combat the HIV epidemic among AYA, and limited evidence of what works, it is important to evaluate novel and potentially scalable strategies to improve HIV outcomes in this population in Sub-Saharan Africa. Importantly, there is a lack of rigorous studies of *any* HCW training programs in resource-limited settings to improve adolescent-friendly services [29, 30]. This study fills several important gaps in AYA HIV research. We are unaware of any other intervention that uses SPs to improve provider skills in working with HIV-positive AYA. While SPs are standard in pre-service medical education in high-resource settings, our use of SPs for an in-service training intervention is novel. This study will measure intervention effectiveness on AYA health outcomes using a rigorous RCT design. This study leverages Kenya’s new EMR system for study outcomes, which is resource-efficient. This study is responsive to the Kenyan Ministry of Health request for novel interventions to inform the implementation of new adolescent-friendly HIV service guidelines [52, 53]. In addition, this study will provide data on core AYA HIV indicators for the UN “90-90-90” targets from a range of geographic and epidemiologic settings in Kenya.

Understanding the cost-effectiveness of HIV interventions for AYA is important for policy-makers to allocate resources effectively [54]. If the SP intervention is effective, we expect to generate novel and useful information on the cost-effectiveness of this intervention. To minimize the costs associated with this intervention, we designed the training to align with the local facility infrastructure, staffing, and training schedules. If the intervention is not effective, we will nonetheless generate estimates of the total cost of this training, as well as the direct and indirect costs to AYA for seeking HIV care, which will be useful for the Kenyan Ministry of Health and future economic analyses of AYA HIV interventions.

Successful implementation of the SP intervention requires careful consideration of several contextual factors. First, strong engagement of stakeholders from NASCOP, county governments, community members, and non-governmental organizations that support facilities is essential. Our established partnerships with NASCOP and county leadership has facilitated access to study facilities and EMR data, and enabled us to stay current on the evolving landscape of adolescent-friendly services in Kenya. Youth perspectives are necessary to include in adolescent health interventions [19]. Our CAB offers important insights into AYA attitudes towards HCWs and challenges of living with HIV. In addition, AYA case scripts, which are the foundation of the training intervention, require multiple revisions to ensure that they are tailored to the local clinical context. Cases were refined through pilot testing with HCWs from a range of cadres and through feedback from SPs, faculty experts, and AYA. Finally, EMR data abstraction is a complex process that likely will vary by partner organization and facility. The EMR system is new in Kenya, and facilities have different capacities for data entry and retrieval. We have established relationships with each partner organization that supports EMR systems at each facility. We can also offer sites support in the data abstraction process.

This study also has limitations. A 3-month strike by public-sector physicians and ensuing staff shortages in public facilities resulted in delays in baseline data collection, and a nursing strike is ongoing. Our ability to recruit AYA for the surveys depends on their school breaks and facilities’ schedules for this patient population. As with any in-service training, turnover of SPEED-trained HCWs is possible, and would dilute the intervention effectiveness. A limitation of a stepped-wedge design is the potential bias introduced by contextual factors that vary over time [32]. We will monitor these changes with the facility survey and evaluate them in the analysis. This intervention targets HCWs to improve AYA retention in HIV care. Other community factors, including HIV-stigma, family and peer support, and school schedules, may also affect retention rates, which we do not capture [4, 11, 14]. Finally, our primary outcomes will depend on the quality of EMR data.

A standardized patient intervention for HCW has the potential to promote high-quality patient-centered HIV services for AYA by providing HCWs with technical skills and pragmatic experiences. This improvement in delivery of care can, in turn, address barriers cited by AYA and improve retention in essential HIV services. By increasing engagement in care, the intervention can ultimately improve quality of life, survival, and decrease subsequent HIV transmission among AYA in Kenya, closing the gap in the “90-90-90” targets.

## Trial registration

Pilot testing of the intervention was completed in October 2016. Participant recruitment is ongoing, and the trial is expected to end in 2020. The trial is registered at ClinicalTrials.gov (NCT02928900).

## Additional files

Additional file 1: Example case script. (EPS 658 kb)

Additional file 2: Example Consent Form, HCW. (PDF 171 kb)

Additional file 3: SPIRIT Checklist. (DOC 122 kb)

## Abbreviations

APS: Adolescent package of services
AYA: Adolescents and Young Adults
AYA: Adolescents and young adults aged 10–24 years
CAB: Community Advisory Board
DALY: Disability-adjusted life year
EAP: External Advisory Panel
EMR: Electronic medical record
GLMM: Generalized linear mixed models
HCW: Healthcare worker
ITT: Intent-to-treat
LTFU: Lost to follow-up
MOH: Ministry of Health
NASCOP: National AIDS and STI Control Program
ODK: Open Data Kit
SP: Standardized patients
SPEED: Simulated Patient Encounters to promote Early Detection and engagement in care for adolescents
