# Supplementary material for: Simulated patient encounters to improve adolescent retention in HIV care in Kenya: study protocol of a stepped-wedge randomized controlled trial
Source: Trials. 2017 Dec 28;18:619. doi: 10.1186/s13063-017-2266-z (PMC5745919; doi:10.1186/s13063-017-2266-z)
Supplement: Supplementary file 2 — Example Consent Form, HCW. (PDF 171 kb) [file 13063_2017_2266_MOESM2_ESM.pdf]

University of Washington (UW) & University of Nairobi (UoN) Collaborative Study Group

Simulated Patient Encounters to Promote Early Detection and Engagement in Care for Adolescents (SPEED)

Consent for Health Care Workers in HIV care to participate in a randomized controlled trial of a clinical training intervention

Investigators

NAME	POSITION	DEPARTMENT	TELEPHONE NUMBERS
Pamela Kohler	Principal Investigator	Global Health, UW	+1-206-616-7962
Dalton Wamalwa	Site Leader	Paediatrics, UoN	020-271-4851 / 0735-558943
Irene Inwani	Co-Investigator	Paediatrics, UoN	0722-608-483
Cyrus Mugo	Study Coordinator	Paediatrics, UoN	0721 599 626
Anjuli Wagner	Co-Investigator	Global Health, UW	+1-978-460-2331
Kate Wilson	Co-Investigator	Global Health, UW	+1-206-618-7417
David Bukusi	Co-Investigator	HIV Prevention Unit, KNH	0721 207 885

Emergency telephone number: Professor Dalton Wamalwa, Department of Paediatrics, University of Nairobi: 0735-558943
Ethics and Research Committee Chairperson: Professor AN Guantai, Telephone 272-6300
University of Washington Human Subjects Division: Telephone +1-206-543-0098.

A. Researchers' statement
1. Introduction

We are asking your consent to volunteer for a research study. The study is being done by the University of Nairobi and the University of Washington. Before you decide whether to take part in the study, we would like to explain the purpose, risks and benefits of the study. We will tell you what we would expect of you if you agree to be in the study. It is important that you understand that it is your choice to participate in this study. You are NOT required to participate in this study to keep your job. Taking part in this study (and any data collected such as performance ratings and written summaries) will not adversely affect your employment in any way. We will not share competency scores with anyone in a way that would be identifiable. This form will help you decide if you want to take part in this study. If you choose to be a part of this study, we will ask you to sign your name or make your mark on this form. We will give you a copy of this form to keep. This process is called informed consent.

It is important that you know the following:

- You do not have to be in this study if you do not want to join.
- If you join the study, you can withdraw from the study at any time without being victimized.
- If you decide not to take part in this study, you can still join another research study later.

2. Purpose and Benefits

The purpose of this study is to evaluate a clinical training intervention to improve quality of care for HIV-positive adolescents (ages 10-24) in Kenya. This study will take place in 24 facilities in Kenya. Health care providers at each facility will participate in a training intervention though role-playing sessions with standardized patient (SP) actors to improve communication skills and competency. A patient actor is a trained actor who will play the role of an HIV-positive adolescent patient to help the health worker to improve skills in counseling and clinical care.

We also would like to better understand whether a clinician training using SP actors can improve adolescent outcomes, including retention in care. Results from this study may be used to improve HIV care and treatment programs for HIV-positive adolescents in Kenya. This study will be carried out in health facilities in Kenya for about three years. Different clinics will receive the training at different times in the three-year period.

3. Procedures

If you agree to participate in this study, you will be asked to take part in a training intervention with SP actors and to have the role-play sessions with actors video-recorded.

Clinical training intervention with SP actors

If you agree to participate in this study, you will be asked to take part in a clinical training intervention. Before the training, you will complete a short survey on your background, training experience, and competency. After completing the baseline survey, you will be asked to take part in a clinical training intervention, with lectures and role-plays. Training will occur in a training facility in your county. This training will last 2-3 days. The training will occur during your normal work hours. During the training, lectures and discussion sessions will cover topics including adolescent development, important issues faced by HIV-positive adolescents, and counseling skills. Patient actors will role-play adolescents who are presenting for HIV care, and you will role-play being their care provider.

For each session, you will be asked to act as you would with a real patient. Providers who participated in this training will have a group de-briefing session with the SP and Study Trainer. Patient actors and the Trainer will provide verbal feedback on your performance. Videos of the patient encounters will also be reviewed and discussed during the debriefing session. The debriefing session should take no more than 90 minutes. At the end of the training, you will be asked to complete a short survey about your satisfaction with the training and your competency. The surveys will take about 10-15 minutes each.

At the end of each training week, a team of experts from UW and UoN will review the videos of the encounters posted on a secure website and rate your performance. You will receive a written summary of their feedback at the end of the training.

After the training intervention, you will be asked to complete a survey about your satisfaction with the training and your competency. The surveys will take about 10-15 minutes each.

In addition, you may be asked to complete periodic surveys on your competency to provide care to HIV-positive adolescents. These surveys will occur approximately every 9 months until the end of the trial. This survey will help to understand if the training was beneficial to you and your adolescent patients. Each survey should take about 10-15 minutes.

You will also be asked to complete an exit interview about one year after your training. The exit interview will ask open-ended questions about your experience in the training, and barriers and facilitators to implementing the skills that you learned. Interviews will be tape-recorded. This exit interview will help to gain a more in-depth understanding of whether the training was beneficial and how it could be improved. The interview will be conducted in a private room and last no more than 30 minutes.

Video-recording of role-plays

All encounters with patient actors will be video-taped. This is an important part of the training approach. The videos will be reviewed by study team members to evaluate your performance in the role-play sessions.

Your participation in this study will end once the trial ends.

4. Risks, Stress, and Discomfort

The encounter with the patient actors might make you feel uncomfortable, because the patient actor may present concerns about sensitive topics like sexual identity, HIV-stigma, violence, and mental health. You also may feel uncomfortable during the debriefing session because you will receive feedback on your performance. You can stop the encounter if you do not want to participate anymore. You can stop the debriefing session at any time if you do not want to participate anymore. You can withdraw your participation at any time.

5. Other information, Confidentiality

The information that you give us will be kept private. We will store video recordings from the encounters in a password protected website. All paper forms will be stored in a room under lock and key. Audio-recordings will be transcribed. Data from the survey and interview will be stored on a password protected server. Only authorized study team members will have access to study records. You will have the option to sign <u>a separate form</u> that would authorize us to use video recordings for education purposes. We will destroy the audio- and video-recording after 6 years. When we publish study results, we will not use your name or identify you personally.

There is no cost to you for being in this study. You will receive light refreshments and transport reimbursement for the intervention training.

You are free to withdraw or refuse to participate in the research study at any time. Your decision to take part in this study or not take part will have no effect on your routine work.

Problems or Questions:

If you ever have any questions about this study, you should contact Dalton Wamalwa at Kenyatta National Hospital (KNH) extension 43560 or 2733087 or 0721239493. If you have questions about your rights as a research participant, or feel that you have been harmed by the study, you should contact Professor Guantai, the Chair of the KNH/UoN Ethics and Research Committee, at Telephone 272-6300. You can also contact the UW Human Subjects Division at +1-206-543-0098.

B. Study Participant's statement

This study has been explained to me. I have had a chance to ask questions. If I have more questions later, I can ask one of the researchers listed above. If I have questions about my rights as a research subject, I can call the KNH/UoN Ethics and Research Committee at 2726300. I will receive a copy of the consent form.

I agree to volunteer to participate in the training intervention ☐

I agree to have the role play sessions video-recorded ☐

______________________	___________________________	_________________
Participant's Name	Signature/Thumbprint	Date
______________________	__________________________	_______________
Study Staff Conducting Consent Discussion	Signature	Date
